# Antenatal care for alcohol consumption during pregnancy: pregnant women’s reported receipt of care and associated characteristics

**DOI:** 10.1186/s12884-019-2436-y

**Published:** 2019-08-16

**Authors:** Emma Doherty, John Wiggers, Luke Wolfenden, Amy E. Anderson, Kristy Crooks, Tracey W. Tsang, Elizabeth J. Elliott, Adrian J. Dunlop, John Attia, Julia Dray, Belinda Tully, Nicole Bennett, Henry Murray, Carol Azzopardi, Melanie Kingsland

**Affiliations:** 1 Hunter New England Population Health, Hunter New England Local Health District, Locked Bag 10, Wallsend, NSW 2287 Australia; 2 School of Medicine and Public Health, The University of Newcastle, Callaghan, New South Wales Australia; 3 Hunter Medical Research Institute, New Lambton Heights, New South Wales Australia; 4 Menzies School of Health Research, Charles Darwin University, Darwin, Northern Territory Australia; 5 Faculty of Medicine and Health and Discipline of Child and Adolescent Health, University of Sydney, Sydney, New South Wales Australia; 6 Sydney Children’s Hospital Network, Kids’ Research Institute, Westmead, New South Wales Australia; 7 Drug and Alcohol Clinical Services, Hunter New England Local Health District, Newcastle, New South Wales Australia; 8 Maternity and Gynaecology John Hunter Hospital, New Lambton Heights, New South Wales Australia

**Keywords:** Maternal, Alcohol consumption, Pregnancy, Antenatal care, Implementation, Evidence-based practice, Quantitative methods

## Abstract

**Background:**

Antenatal clinical guidelines recommend that during initial and subsequent antenatal visits all pregnant women: have their alcohol consumption assessed; be advised that it is safest not to consume alcohol during pregnancy and of the potential risks of consumption; and be offered referrals for further support if required. However, the extent to which pregnant women attending public antenatal services receive guideline recommended care at these visits, and the characteristics associated with its receipt, is unknown. The purpose of this study was to examine: 1) pregnant women’s reported receipt of guideline recommended care addressing alcohol consumption during pregnancy; 2) characteristics associated with the receipt of care; and 3) pregnant women’s acceptability of care.

**Methods:**

From July 2017 – February 2018 a survey (telephone or online) was undertaken with 1363 pregnant women who had recently visited a public antenatal service in one health district in Australia. Receipt and acceptability of recommended care were assessed via descriptive statistics and associations via logistic regression analyses.

**Results:**

At the initial antenatal visit, less than two thirds (64.3%) of pregnant women reported that they received an assessment of their alcohol consumption and just over one third (34.9%) received advice and referral appropriate to their self-reported level of alcohol consumption since pregnancy recognition. Less than 10% of women received such care at subsequent antenatal visits. Characteristics that significantly increased the odds of receiving all guideline elements at the initial antenatal visit included: less than university attainment (OR = 1.93; 95% CI:1.12, 3.34), not residing in an advantaged area (OR = 2.11; 95% CI:1.17, 3.79), first pregnancy (OR = 1.91; 95% CI:1.22, 2.99) and regional/rural service location (OR = 2.38; 95% CI:1.26, 4.48); and at subsequent visits: younger age (OR = 0.91; 95% CI:0.84, 0.99) and Aboriginal origin (OR = 3.17; 95% CI:1.22, 8.24). Each of the recommended care elements were highly acceptable to pregnant women (88.3–99.4%).

**Conclusions:**

Although care for alcohol consumption is both recommended by clinical guidelines and highly acceptable to pregnant women, its receipt in public antenatal services is suboptimal. There is a need and an opportunity for interventions to support antenatal care providers to routinely and consistently provide such care to all pregnant women.

## Background

Prenatal alcohol exposure is recognised as a risk factor for a number of adverse pregnancy outcomes including spontaneous abortion, stillbirth, preterm birth, fetal growth restriction and low birth weight, and can result in lifelong cognitive, behavioural and neurodevelopmental disabilities for the child [1]. Although the fetus is most vulnerable to structural damage due to the effects of alcohol exposure in the first trimester [2], exposure to alcohol throughout the duration of pregnancy has been associated with poorer pregnancy outcomes [3]. On this basis, many countries, including Australia, have issued national guidelines recommending that it is safest for women who are pregnant or planning a pregnancy not to consume alcohol [1, 4, 5].

Despite such guidelines, approximately 10% of women globally consume alcohol at any time during pregnancy, with higher prevalence estimates reported in countries with high alcohol consumption rates in the general population (e.g. Ireland: 60%; Denmark: 46%; United Kingdom: 41%) [6]. In Australia, prospective cohort studies and national surveys have reported the prevalence of alcohol consumption at various times during pregnancy to be between 28 and 72% [7–14]. For example, a prospective cohort study of 1570 pregnant women found that 59% of women reported any alcohol consumption during pregnancy with 32% reporting consumption in the second and/or third trimester [10]. Similarly, a national survey conducted in 2016 found that half of pregnant women consumed alcohol before knowing they were pregnant and 25% continued consuming alcohol following knowledge of their pregnancy [11]. Among those women who consumed alcohol during pregnancy, most reported drinking at a frequency of monthly or less (81%) and an average of one to two standard drinks per occasion (97%) [11].

Systematic review evidence indicates that brief interventions delivered by a range of health professional groups (e.g. general practitioners, specialists, nurses and psychologists) are effective in reducing alcohol consumption in patients attending general practice, primary care and hospital emergency settings [15, 16]. Review evidence from primary care settings also suggests that such brief interventions are cost-effective [17]. For pregnant women specifically, psychological, educational and brief interventions have been reported to be effective in increasing abstinence from alcohol and modifying alcohol consumption behaviours during pregnancy [16, 18].

Given such findings and the potential adverse outcomes associated with prenatal alcohol exposure, international [19] and Australian [20–22] antenatal clinical guidelines recommend that all pregnant women be asked about their alcohol consumption using a validated assessment tool and be advised that it is safest not to consume alcohol during pregnancy and of the potential risks associated with consumption. For pregnant women identified as currently consuming alcohol at levels where they may find it difficult to abstain, it is recommended that referrals be offered to therapeutic support services or drug and alcohol services for specialist assessment and treatment [19–22]. It is recommended that this assessment and care be provided by the attending antenatal care provider at the initial antenatal visit as well as in subsequent antenatal visits [19–22]. The aim of such guidelines therefore are to encourage women who have not consumed alcohol since pregnancy recognition to continue abstaining for the remainder of their pregnancy and to accurately identify women who are currently consuming alcohol so that appropriate support can be offered.

In countries that have widespread use of publicly funded health care, such as Australia and the United Kingdom, public antenatal services are a critical setting for these guideline recommendations to be implemented. For instance, in Australia 70% of women access public antenatal care at some stage throughout their pregnancy, with 55% using an exclusive public antenatal model of care from the point of booking in with the hospital at approximately 14 weeks gestation [23]. Such services cater to a diverse range of population groups, including the most vulnerable, and generally have contact with pregnant women on multiple occasions throughout pregnancy to be able to monitor and respond to risks [24].

Despite public antenatal services being a critical setting, the extent to which pregnant women receive all care elements aligned with current antenatal clinical guideline recommendations at both initial and subsequent antenatal visits in these services is unknown. Internationally, the majority of existing studies describing antenatal care for maternal alcohol consumption have not been specific to the public antenatal setting, but rather have focussed on care provision by a range of health professionals (e.g. general practitioners, obstetrician-gynaecologists, paediatricians, midwives, community nurses and allied health) [25–28] or have not defined the setting in which care was received [29–37]. Of the six studies identified specific to the public antenatal setting [38–43], none have reported the prevalence of the individual elements of recommended care (assessment, advice, referral) received across multiple visits (initial and subsequent visits) [19–22]. In addition, half of existing studies have used self-report measures of care provision by antenatal care providers [39–41], which can result in an overestimate of care delivery. Client self-report has been suggested as a recommended approach when measuring clinical guideline adherence, as although it may produce more conservative results than clinician self-report, it is subject to less response bias [44].

Studies reporting antenatal care provision in public antenatal services suggests that it is highly variable. For instance, a survey of 103 Norwegian midwives found that 97% mostly or always ask about alcohol consumption at the initial antenatal visit (42% via a validated tool) and 66% mostly or always provide a referral to the woman’s general practitioner when risky alcohol consumption is identified [40]. A study of 439 Danish pregnant women found that about half (51%) reported being asked about alcohol consumption and 11% advised that it is safest not to consume alcohol during pregnancy [38], however, the study did not define the visit in which care was received. In an Australian study of 223 pregnant women, 92% reported being asked about alcohol and, of those women who reported consuming alcohol during pregnancy, 10% were offered assistance to manage their alcohol consumption [43]. The study did not report whether the questions women received were consistent with a validated assessment tool. Such studies echo the broader literature in a range of health care settings, which has found that appropriate care in response to screening is often not provided [45]. Given the limitations of existing studies, it is unknown whether current public antenatal care for alcohol consumption during pregnancy aligns with guideline recommendations at both initial and subsequent antenatal visits.

Given the recommendation that assessment and care for alcohol consumption during pregnancy is routinely undertaken with all women, there is also a need to assess if current care is being delivered consistently to all women irrespective of their characteristics or those of the antenatal service. Previous studies have found that women who are younger, do not have a university degree, are of a minority ethnicity, are attending antenatal care at a smaller centre [27] or seeing a midwife (as opposed to a doctor) [46] are more likely to receive alcohol assessment and advice from their antenatal care provider [34]. Whereas, consistent with the universal screening recommendations of antenatal clinical guidelines, women’s actual alcohol consumption behaviours during pregnancy have not been found to be associated with being asked about alcohol consumption [38, 47]. Studies examining adherence to antenatal care guidelines more broadly have also found that women who have had a previous pregnancy and do not have a history of pregnancy complications [48] are more likely to receive guideline care, but the evidence is mixed [43, 49, 50]. No studies have examined the characteristics associated with the receipt of all guideline elements for assessment and care for alcohol consumption during pregnancy at initial and subsequent visits. In the absence of such information, it is unknown whether all women have the same opportunity to receive recommended care for alcohol consumption during pregnancy.

There is also a need to assess pregnant women’s acceptability of guideline recommended care for alcohol consumption as this may be an impediment to antenatal care providers delivering such care routinely to all women. Currently, limited studies have examined women’s acceptability for each of the care elements recommended by antenatal clinical guidelines [19–22]. For instance, in one Australian study conducted with 1103 women, nearly all women agreed that antenatal care providers should ask pregnant women about their alcohol consumption (97%) and advise pregnant women to abstain from consuming alcohol (91%) [51]. However, the study did not include women who were currently pregnant and did not assess whether acceptability varied by women’s alcohol consumption behaviours. Therefore, an assessment of pregnant women’s acceptability is required to determine whether it is a potential barrier to routine guideline care provision.

Given the current gaps in evidence, this study was undertaken to examine: 1) pregnant women’s reported receipt of antenatal clinical guideline recommended care (assessment, advice and referral) for alcohol consumption during pregnancy at their initial antenatal visit and subsequent antenatal visits; 2) associations between the characteristics of pregnant women and antenatal services and the receipt of recommended care in these visits; and 3) pregnant women’s acceptability of such care.

## Methods

### Ethics approval

The study was approved by the Hunter New England Human Research Ethics Committee (16/11/16/4.07), Aboriginal Health and Medical Research Council (1236/16) and the University of Newcastle Human Research Ethics Committee (H-2017-0032).

### Design and setting

A cross sectional survey of pregnant women attending antenatal care in three sectors within a health district in New South Wales, Australia was undertaken from July 2017 to February 2018. The services provide public antenatal care to 70% (over 6000 annually) of women giving birth in the district’s public hospitals in metropolitan, regional and rural locations.

### Participants and recruitment

#### Public antenatal services

All (*n* = 5) public antenatal services within the study area were included in the study. Such services provide a range of antenatal care models, including hospital and community-based midwifery clinics, midwifery group practice continuity of care, specialist medical clinics, Aboriginal Maternal Infant Health Services (AMIHS) and multidisciplinary care for women with complex pregnancies or identified vulnerabilities. Care is provided by registered midwives, medical practitioners, Aboriginal Health Workers and students and is supported by a range of other professions, such as social workers. The number and type of antenatal care providers present in each antenatal visit differs by care model.

#### Pregnant women

Women attending any of the public antenatal services within the study area were eligible to participate in the study if they: were at least 18 years of age; were between 12 and 37 weeks gestation; and had attended a face-to-face antenatal visit in the preceding week for either an initial antenatal visit or a visit between 27 and 28 weeks or 35 and 36 weeks gestation (inclusive). Women were deemed ineligible if either: their antenatal care was through a private obstetrician; they had already been selected to participate in the survey in the past 4 weeks; they had previously declined participation in the survey; or they had given birth or had a negative pregnancy outcome (stillbirth or miscarriage).

#### Recruitment procedure

All women received written information at their first antenatal visit informing them about the survey and that they might be sampled throughout their antenatal care based on their attendance at the service. The information provided included a toll free telephone number that women could call to register that they did not want to be sampled for the study. Electronic medical record and appointment data were used to generate a weekly sample of eligible women across the five public antenatal services as a group. From an average of 188 women per week who had an appointment, 150 were on average eligible for sampling. From these eligible women, 105 (initial antenatal visit: 30; 27–28 weeks gestation: 30; 35–36 weeks gestation: 45) were randomly selected via a computerised random-number generator and mailed an information statement outlining the purpose of the survey and inviting them to participate. In the information statement women were informed that the study team had not had direct access to their medical records and only information required to invite participation had been provided by the antenatal service. One week later, non-Aboriginal women were followed-up by telephone and invited to participate in a computer assisted telephone interview (CATI). Based on advice received regarding a culturally appropriate survey approach and as per formal ethics approval, women of Aboriginal or Torres Strait Islander origin and/or women attending or enrolled to attend an AMIHS received a text message after the information statement was mailed and provided the option of completing the survey via CATI or online. Those women who chose to complete the survey online, provided their written consent to participate via text message. Consent was obtained via text message to reduce participant burden by only sending an online survey link to those women who provided their consent to participate in the study. The online survey link was sent to the participant within 48 h of consent being obtained and was unique to the participant to provide ease of access to the survey and to allow data protection. A reminder that participation was voluntary and that it was possible to decline participation at any stage by not submitting a completed survey was provided on the first screen of the online survey prior to the woman entering into the survey. Women’s consent and online survey completion status were saved in the survey database. Women who did not respond to the text were followed up with a telephone call 4 days later and invited to participate in the survey.

As per formal ethics approval, women who received a telephone call inviting participation gave their verbal consent to participate, which was recorded by the CATI interviewer into the survey database prior to the commencement of the survey. Eligibility related to English language proficiency (sufficient to complete the survey unaided) was also assessed at the beginning of the CATI. Women were given the opportunity to decline survey participation at the point of receiving the information statements (via a toll free number), the text message or at any stage during the survey. All women who declined participation in the CATI were provided the option to complete the survey online. Women received up to 10 phone contact attempts within a 2 week period. Women who chose to complete the survey online were asked to complete the survey within the same two-week period. As per ethics requirements, medical records were checked by a local health district staff member prior to women being called and any women who had given birth or had a negative pregnancy outcome were made ineligible for participation in the study.

### Data collection procedures

The survey questions were developed based on previous Australian national and state surveys [11, 42] and surveys conducted in health care settings to assess patient self-report of care receipt [52–54]. The online survey was developed using REDCap [55] and was accessible via email or text message using a unique survey link. CATI surveys were undertaken by trained and experienced female interviewers. The online and CATI surveys were reviewed for cultural appropriateness for Aboriginal and Torres Strait Islander women and pilot tested prior to use. Data regarding antenatal service characteristics were obtained from electronic medical record and appointment systems and linked to individual participant data from the CATI and online surveys.

### Measures

#### Pregnant women’s alcohol consumption since pregnancy recognition

All women were asked to report their alcohol consumption since pregnancy recognition using the three item AUDIT-C screening tool (how often have a drink containing alcohol, how many standard drinks consumed on a typical drinking day and how often five or more standard drinks consumed on one occasion) [56]. A systematic review of brief alcohol screening instruments in pregnancy found the AUDIT-C to have the highest sensitivity for identifying risky alcohol consumption among pregnant women [57]. Australian national guidelines classify ‘medium risk of harm’ in pregnancy as an AUDIT-C score of three to four and ‘high risk of harm’ in pregnancy as an AUDIT-C score of five plus [58]. Women at medium and high risks of harm are likely to require further support to abstain from alcohol during pregnancy [58].

#### Receipt of antenatal care for maternal alcohol consumption

All women completed survey items assessing whether they were asked any questions by their antenatal care provider/s about their alcohol consumption during the antenatal visit and, if so, whether they were asked questions consistent with the AUDIT-C [56] (were you asked: how often you currently consume alcohol; number of standard drinks on a typical drinking day; and occasions of consuming 5 or more standard drinks) (possible responses: yes, no, don’t know). All women were also asked whether they were advised that it is safest not to consume alcohol during pregnancy; advised of the potential risks associated with consuming alcohol during pregnancy; and whether they were offered a referral to assist them in managing their alcohol consumption (possible responses: yes, no, don’t know). Women completing the survey for the 27–28 or 35–36 week gestation visits were also asked if they had accepted a referral for managing their alcohol consumption in any other antenatal care visit and, if so, whether the antenatal care provider followed up or discussed the progress of any previously accepted referrals (possible responses: yes, no, don’t know).

#### Characteristics associated with receipt of care

Data were collected on the following characteristics of pregnant women and antenatal services that were identified as potentially associated with provision of antenatal care to address maternal alcohol consumption during pregnancy [27, 34, 38, 46–48].

- Pregnant women’s characteristics. Women reported: whether they were of Aboriginal or Torres Strait Islander origin; their age; their highest level of education completed; whether this was their first pregnancy; and whether they had consumed alcohol since pregnancy recognition [56]. Women’s allocated model of antenatal care (hospital and community-based midwifery clinic, specialist medical clinic, midwifery group practice continuity of care, multidisciplinary care for women with complex medical needs, AMIHS, multidisciplinary care for women with identified vulnerabilities) and residential postal code were obtained from the electronic medical record and appointment systems.
- Antenatal service characteristics. The antenatal service’s postal code was obtained from the electronic medical records and women reported the type of antenatal care provider seen in their visit with the service (possible responses: midwife, hospital doctor (e.g. specialist obstetrician, registrar), Aboriginal Health Worker, other, don’t know).

#### Acceptability of antenatal care for maternal alcohol consumption

Women’s acceptability of receiving assessment and care for alcohol consumption during antenatal care visits was assessed using a 5-point Likert scale (possible responses: strongly agree, agree, unsure, disagree, strongly disagree) and were informed by previous surveys with patients attending a health service [54]. Women reported whether during their antenatal care it was acceptable to be: asked about their alcohol consumption; asked about their alcohol consumption on multiple occasions; advised that it is safest not to consume alcohol during pregnancy; advised of the potential risks associated with alcohol consumption during pregnancy; offered referral to a telephone based counselling service for further support if required; and offered referral to the health district’s Drug and Alcohol clinical service for further support if required.

### Statistical analyses

All statistical analyses were undertaken using SAS version 9.3 [59]. Condensed response categories were created for women’s Aboriginal and Torres Strait Islander origin (‘Aboriginal or Torres Strait Islander or both’ or ‘Neither Aboriginal or Torres Strait Islander’), highest education level completed (‘Completed high school or less’ or ‘Completed technical certificate or diploma’ or ‘Completed university or college degree or higher’), women’s self-reported alcohol consumption since pregnancy recognition (‘yes’ (for AUDIT-C score ≥ 1) or ‘no’ (for AUDIT-C score of 0)) and antenatal care providers seen in the visit (‘midwife only’ or ‘doctor only’ or ‘midwife and doctor’ or ‘other provider involved’). Antenatal visits at 27–28 and 35–36 weeks gestation were also condensed to create a ‘subsequent antenatal visits’ variable. Women’s allocated model of antenatal care was used to indicate pregnancy risk level, with hospital and community-based midwifery clinics, midwifery group practice continuity of care and multidisciplinary care for women with social vulnerabilities used to classify ‘low risk pregnancy’ and specialist medical clinics and multi-disciplinary care for women with complex medical needs models used to classify ‘high risk pregnancy’. Women’s residential postal codes were used to determine socio-economic disadvantage using the Index of Relative Socio-Economic Disadvantage (IRSD) [60] with index quintiles collapsed into ‘most disadvantaged’ (quintiles one and two), ‘mid disadvantaged’ (quintile three) and ‘least disadvantaged’ (quintiles four and five). Antenatal service postal code was used to calculate the antenatal service’s geographical remoteness (‘major city’ or ‘regional or rural’) using the Access/Remoteness Index of Australia [61]. Women’s reported acceptability of each of the care elements was dichotomised into ‘acceptable’ (strongly agree and agree) and ‘not acceptable’ (strongly disagree, disagree and unsure).

The following assessment and care delivery outcome variables were created:

- ‘assessment (AUDIT-C)’: reported receipt of a question consistent with the first AUDIT-C question (for women who reported in the survey that they had not consumed alcohol since pregnancy recognition (i.e. AUDIT-C score = 0)) and reported receipt of all three questions consistent with the AUDIT-C (for women who reported in the survey that they had consumed alcohol since pregnancy recognition (i.e. AUDIT-C score ≥ 1)).
- ‘complete advice’: reported receipt of advice that it is safest not to consume alcohol during pregnancy and of the potential risks associated with alcohol consumption during pregnancy (for all women regardless of their alcohol consumption since pregnancy recognition).
- ‘referral offered or followed up’: reported receipt of referral offer or follow up for women who reported in the survey that they had consumed alcohol at medium or high risk levels since pregnancy recognition (i.e. AUDIT-C score: ≥ 3).
- ‘complete care’: reported receipt of complete advice (all women) and referral offered or followed up (for women who reported in the survey an AUDIT-C score ≥ 3).
- ‘all guideline recommended elements’: reported assessment via AUDIT-C (all women) and complete advice (all women) and referral offered or followed up (for women who reported in the survey an AUDIT-C score ≥ 3).

Descriptive statistics were used to describe pregnant women and antenatal service characteristics; receipt of assessment and care; and acceptability of care. Pregnant women’s acceptability of care was also assessed for women who had consumed alcohol since pregnancy recognition as a subgroup analysis. Associations between maternal and service characteristics and the receipt of antenatal care for maternal alcohol consumption were assessed using bivariate and multivariable logistic regression analyses. Bivariate analyses (chi square for categorical variables and t-test for continuous variables) were first undertaken to test the individual associations between each of the characteristics with the receipt of three care elements (assessment (AUDIT-C), complete care, and all guideline recommended elements) at the initial antenatal visit and subsequent antenatal visits. The variable ‘consumption of alcohol since pregnancy recognition’ was not included in any association analyses for the initial antenatal visit as the attending antenatal care provider would not routinely have prior knowledge of a woman’s alcohol consumption and as such it is not hypothesised to be associated with the provision of assessment and care at this visit. Multivariable logistic regression analyses were then undertaken to test the associations between all the characteristics with the receipt of three care elements (assessment (AUDIT-C), complete care, and all guideline recommended elements) at the initial antenatal visit and subsequent antenatal visits.

## Results

### Participants

All (*n* = 5) public antenatal services in the study area participated. A total of 2840 eligible women were sent an information letter and invited to participate in the survey. On the day of contact, 2546 (90%) women were deemed eligible to participate based on electronic medical record data that they had not given birth or had a negative pregnancy outcome since being sampled or had not refused participation via the toll free number. Of the 1768 (62%) women who were able to be contacted within the 2 week period, 1712 (97%) were deemed eligible to participate based on English language proficiency and having not given birth or experienced a negative pregnancy outcome as reported by the woman. Of these, 1397 (82%) women consented to participate and 1363 (80%) completed the survey. A lower proportion of Aboriginal and Torres Strait Islander women compared to non-Aboriginal women consented to participate in the survey (58% vs 84%, *p* < .001). Pregnant women and antenatal service characteristics are reported in Table 1.

**Table 1:** Characteristics of pregnant women and antenatal services (*n* = 1363)

Characteristic	N (%)
Age	
Mean (SD)	29 years (5 years)
Aboriginal, or Torres Strait Islander, or both	80 (6%)
Highest education level completed	
Completed high school or less	398 (29%)
Completed technical certificate or diploma	496 (36%)
Completed university or college degree or higher	469 (34%)
Area index of disadvantage	
Most disadvantaged	574 (42%)
Mid disadvantaged	454 (33%)
Least disadvantaged	335 (25%)
First Pregnancy	571 (42%)
Pregnancy risk level	
Low risk	851 (62%)
High risk	512 (38%)
Consumed alcohol since pregnancy recognition (yes)	133 (10%)
Antenatal service geographic remoteness	
Major city	1146 (84%)
Regional or rural	217 (16%)
Provider/s seen in antenatal visit	
Midwife only	821 (60%)
Doctor only	198 (15%)
Midwife and doctor	298 (22%)
Other provider involved	46 (3%)

### Receipt of assessment and care for maternal alcohol consumption in antenatal visits

#### Asked about alcohol consumption and assessment consistent with AUDIT-C

As shown in Table 2, the majority of participants reported being asked about their alcohol consumption (88.8%) and receiving questions consistent with the AUDIT-C assessment (64.3%) at their initial antenatal visit. Significantly lower proportions of women reported being asked about alcohol consumption (14.3%) and assessed via the AUDIT-C (7.8%) at subsequent antenatal visits (*p* < 0.001).

**Table 2:** Pregnant women’s reported receipt of assessment and care for maternal alcohol consumption at initial and subsequent antenatal visits

Element of care reportedly received	Initial antenatal visit (*N* = 473)			Subsequent antenatal visits (*N* = 890)			Comparison between care at initial and subsequent antenatal visits	
	n	%	95% CI	n	%	95% CI	Adjusted Odds Ratio (95% CI)^a^	*p*-value
Asked about alcohol consumption	420	88.8	85.63–91.33	127	14.3	12.13–16.72	47.61 (33.82–67.02)	*p* < 0.001
Assessment (AUDIT-C)	304	64.3	59.95–68.59	69	7.8	6.17–9.70	21.40 (15.71–29.16)	*p* < 0.001
Advised safest not to consume	299	63.2	58.86–67.56	141	15.8	13.59–18.39	9.13 (7.04–11.83)	*p* < 0.001
Advised of potential risks	182	38.5	34.09–42.87	187	21.0	18.46–23.81	2.35 (1.84–3.01)	*p* < 0.001
Complete advice (safest not to consume and potential risks)	166	35.1	30.79–39.40	78	8.8	7.08–10.80	5.63 (4.17–7.59)	*p* < 0.001
Referral offered or followed up^b^	1	50.0	0.00–100.00	0	0.0	0.00–0.00	–	–
Complete care (complete advice and referral offered or followed up)	165	34.9	30.58–39.18	77	8.7	6.98–10.68	5.66 (4.19–7.64)	*p* < 0.001
All guideline elements (assessment, complete advice and referral offered or followed up)	132	27.9	23.86–31.95	34	3.8	2.75–5.29	9.75 (6.55–14.50)	*p* < 0.001

^a^Subsequent visit as Referent

^b^Limited to women who reported in the survey that they had consumed alcohol at medium or high risk of harm levels since pregnancy recognition (AUDIT-C score ≥ 3) (Initial antenatal visit *n* = 2; Subsequent visit *n* = 4). Not included in ‘comparison between care at initial and subsequent antenatal visits’ *p*-value test due to small sample size

#### Advice

Nearly two thirds of participants (63.2%) reported being advised at their initial antenatal visit that it is safest not to consume alcohol during pregnancy, 38.5% reported being advised of the potential risks associated with alcohol consumption during pregnancy and 35.1% reported receiving both components of recommended advice (complete advice). At subsequent antenatal visits, significantly lower proportions of women reported receipt of advice that it is safest not to consume alcohol during pregnancy (15.8%), potential risks associated with alcohol consumption during pregnancy (21.0%) and complete advice (8.8%) (*p* < 0.001).

#### Referral offered or followed up

Two of the participants surveyed after the initial antenatal visit reported that they had consumed alcohol at medium or high risk levels (i.e. AUDIT-C ≥ 3) since pregnancy recognition. One of these participants reported that she was offered a referral for further support to address her alcohol consumption. Four women surveyed after subsequent antenatal visits reported that they had consumed alcohol at medium or high risk levels, with none reporting being offered a referral or having a previously accepted referral followed up in their antenatal visit.

#### Complete care (advice and referral)

At the initial antenatal visit approximately one third of participants (34.9%) reported receiving complete care for alcohol consumption during pregnancy relative to their self-reported alcohol risk level since pregnancy recognition. At subsequent antenatal visits provision of complete care was significantly lower (8.7%, *p* < 0.001).

#### All guideline elements (assessment, advice and referral)

At the initial antenatal visit just over a quarter (27.9%) of participants reported being assessed consistent with the AUDIT-C, receiving advice and being offered a referral or having a previously accepted referral followed up if at medium or high risk. At subsequent antenatal visits significantly lower proportion of women reported receiving all guideline care elements relative to their identified risk level (3.8%, *p* < 0.001).

### Association between receipt of assessment and care for maternal alcohol consumption and characteristics of pregnant women and antenatal services

All care elements both at the initial and subsequent antenatal visits were found to have characteristics associated with reported care receipt (Tables 3 and 4). Adjusting for all characteristics, attending antenatal care at a regional or rural location compared to a major city significantly increased the odds of receiving assessment (OR = 2.74; 95% CI: 1.40, 5.33), complete care (OR = 2.04; 95% CI: 1.10, 3.77) and all guideline recommended elements (OR = 2.38; 95% CI: 1.26, 4.48) at the initial antenatal visit. Additionally, completing high school or less or a technical certificate or diploma increased the odds of reporting complete care (OR = 1.82; 95% CI: 1.05, 3.16; OR = 1.82; 95% CI: 1.09, 3.03) and all guideline elements (OR = 1.88; 95% CI: 1.04, 3.40; OR = 1.93; 95% CI: 1.12, 3.34) at the initial antenatal visit. Being a woman’s first pregnancy was also significantly associated with receiving complete care (OR = 1.78; 95% CI: 1.16, 2.72) and all guideline elements (OR = 1.91; 95% CI: 1.22, 2.99) at the initial antenatal visit.

**Table 3:** Bivariate and multivariable associations between reported receipt of assessment (AUDIT-C), complete care and all guideline elements and maternal and antenatal service characteristics at the initial antenatal visit (*N* = 473)

Characteristic	Assessment (AUDIT-C)					Complete Care (complete advice and referral offered or followed up)					All guideline elements (assessment, complete advice and referral offered or followed up)				
	n (%)	Bivariate Unadjusted OR (95% CI)	*p*-value	Multivariable Adjusted OR (95% CI)	*p*-value	n (%)	Bivariate Unadjusted OR (95% CI)	*p*-value	Multivariable Adjusted OR (95% CI)	*p*-value	n (%)	Bivariate Unadjusted OR (95% CI)	*p*-value	Multivariable Adjusted OR (95% CI)	*p*-value
Age	29 years (SD: 5 years)	0.97 (0.94–1.01)	0.13	0.98 (0.94–1.02)	0.41	28 years (SD: 6 years)	0.92 (0.89–0.96)	< 0.001	0.96 (0.92–1.00)	0.04	28 years (SD: 6 years)	0.94 (0.90–0.98)	< 0.01	0.97 (0.93–1.02)	0.23
Aboriginal or Torres Strait Islander origin or both			0.27		0.53			< 0.01		0.04			0.05		0.20
Yes	18 (75.0%)	1.71 (0.67–4.39)		1.37 (0.52–3.61)		15 (62.5%)	3.32 (1.42–7.77)		2.59 (1.06–6.31)		11 (45.8%)	2.29 (1.00–5.26)		1.78 (0.74–4.28)	
No (referent)	286 (63.7%)					150 (33.4%)					121 (27.0%)				
Education level			0.08		0.17			< 0.01		0.04			< 0.01		0.04
Completed high school certificate or less	98 (68.1%)	1.61 (1.00–2.59)		1.50 (0.89–2.52)		62 (43.1%)	2.42 (1.47–3.98)		1.82 (1.05–3.16)		49 (34.0%)	2.27 (1.33–3.87)		1.88 (1.04–3.40)	
Completed technical certificate or diploma	120 (67.4%)	1.56 (1.00–2.45)		1.50 (0.94–2.40)		67 (37.6%)	1.93 (1.19–3.12)		1.82 (1.09–3.03)		55 (30.9%)	1.96 (1.17–3.30)		1.93 (1.12–3.34)	
Completed university or college degree or higher (referent)	86 (57.0%)					36 (23.8%)					28 (18.5%)				
Area index of disadvantage			0.14		0.14			0.20		0.30			0.05		0.03
Most disadvantaged	132 (66.3%)	1.49 (0.94–2.37)		1.02 (0.61–1.71)		76 (38.2%)	1.55 (0.96–2.52)		1.15 (0.65–2.03)		60 (30.2%)	1.78 (1.04–3.05)		1.28 (0.69–2.39)	
Mid disadvantaged	102 (67.6%)	1.58 (0.96–2.58)		1.55 (0.93–2.58)		54 (35.8%)	1.40 (0.84–2.34)		1.51 (0.87–2.60)		48 (31.8%)	1.92 (1.10–3.37)		2.11 (1.17–3.79)	
Least disadvantaged (referent)	70 (56.9%)					35 (28.5%)					24 (19.5%)				
First pregnancy			0.89		0.67			< 0.01		< 0.01			< 0.01		< 0.01
Yes	124 (63.9%)	0.97 (0.66–1.43)		0.91 (0.60–1.38)		84 (43.3%)	1.87 (1.27–2.74)		1.78 (1.16–2.72)		69 (35.6%)	1.89 (1.26–2.84)		1.91 (1.22–2.99)	
No (referent)	180 (64.5%)					81 (29.0%)					63 (22.6%)				
Pregnancy risk level			0.20		0.49			0.02		0.04			0.06		0.12
Low risk pregnancy	282 (65.1%)	1.53 (0.79–2.94)		1.28 (0.63–2.61)		158 (36.5%)	2.71 (1.17–6.27)		2.60 (1.05–6.44)		126 (29.1%)	2.33 (0.95–5.68)		2.13 (0.82–5.53)	
High risk pregnancy (referent)	22 (55.0%)					7 (17.5%)					6 (15.0%)				
Antenatal service location			< 0.01		< 0.01			0.01		0.02			< 0.01		< 0.01
Regional or rural	57 (78.1%)	2.21 (1.22–3.98)		2.74 (1.40–5.33)		35 (48.0%)	1.91 (1.15–3.17)		2.04 (1.10–3.77)		30 (41.1%)	2.04 (1.22–3.42)		2.38 (1.26–4.48)	
Major city (referent)	247 (61.8%)					130 (32.5%)					102 (25.5%)				
Provider/s seen in antenatal visit			0.23		0.14			0.22		0.50			0.49		0.71
Midwife only	236 (66.5%)	1.62 (0.65–4.02)		1.99 (0.76–5.17)		128 (36.1%)	0.56 (0.23–1.39)		0.53 (0.20–1.42)		103 (29.0%)	0.76 (0.29–1.96)		0.74 (0.27–2.02)	
Midwife and doctor	47 (56.0%)	1.04 (0.39–2.77)		1.21 (0.44–3.38)		23 (27.4%)	0.38 (0.14–1.02)		0.44 (0.15–1.29)		18 (21.4%)	0.51 (0.18–1.46)		0.56 (0.18–1.71)	
Other provider involved	10 (71.4%)	2.05 (0.48–8.77)		3.11 (0.68–14.22)		4 (28.6%)	0.40 (0.09–1.71)		0.41 (0.08–1.98)		4 (28.6%)	0.74 (0.17–3.26)		0.87 (0.18–4.29)	
Doctor only (referent)	11 (55.0%)					10 (50.0%)					7 (35.0%)

**Table 4:** Bivariate and multivariable associations between reported receipt of assessment (AUDIT-C), complete care and all guideline elements and maternal and antenatal service characteristics at subsequent antenatal visits (*N* = 890)

Characteristic	Assessment (AUDIT-C)					Complete Care (complete advice and referral offered or followed up)					All guideline elements (assessment, complete advice and referral offered or followed up)				
	n (%)	Bivariate Unadjusted OR (95% CI)	*p*-value	Multivariable Adjusted OR (95% CI)	*p*-value	n (%)	Bivariate Unadjusted OR (95% CI)	*p*-value	Multivariable Adjusted OR (95% CI)	*p*-value	n (%)	Bivariate Unadjusted OR (95% CI)	*p*-value	Multivariable Adjusted OR (95% CI)	*p*-value
Age	28 years (SD: 6 years)	0.95 (0.91–1.00)	< 0.05	0.98 (0.93–1.04)	0.57	27 years (SD: 6 years)	0.88 (0.84–0.92)	< 0.001	0.91 (0.86–0.96)	< 0.01	26 years (SD: 5 years)	0.88 (0.82–0.94)	< 0.001	0.91 (0.84–0.99)	0.03
Aboriginal or Torres Strait Islander origin or both			< 0.01		0.01			< 0.001		0.02			< 0.001		0.02
Yes	11 (19.6%)	3.27 (1.61–6.66)		2.70 (1.23–5.92)		14 (25.0%)	4.08 (2.11–7.87)		2.40 (1.15–5.04)		8 (14.3%)	5.18 (2.23–12.05)		3.17 (1.22–8.24)	
No (referent)	58 (7.0%)					63 (7.6%)					26 (3.1%)				
Education level			< 0.01		0.04			< 0.001		0.07			0.02		0.23
Completed high school certificate or less	31 (12.2%)	2.32 (1.26–4.25)		1.96 (1.01–3.80)		36 (14.2%)	3.12 (1.69–5.76)		2.01 (1.00–4.01)		17 (6.7%)	3.19 (1.30–7.81)		1.84 (0.67–5.05)	
Completed technical certificate or diploma	20 (6.3%)	1.12 (0.58–2.16)		0.97 (0.49–1.94)		25 (7.9%)	1.61 (0.84–3.08)		1.17 (0.58–2.34)		10 (3.1%)	1.44 (0.54–3.84)		0.94 (0.33–2.68)	
Completed university or college degree or higher (referent)	18 (5.7%)					16 (5.0%)					7 (2.2%)				
Area index of disadvantage			0.39		0.43			0.69		0.97			0.43		0.69
Most disadvantaged	33 (8.8%)	1.61 (0.81–3.19)		1.61 (0.76–3.40)		36 (9.6%)	1.22 (0.67–2.23)		1.07 (0.53–2.15)		18 (4.8%)	1.48 (0.61–3.59)		1.41 (0.51–3.84)	
Mid disadvantaged	24 (7.9%)	1.43 (0.70–2.93)		1.50 (0.72–3.14)		24 (7.9%)	0.99 (0.52–1.89)		1.00 (0.50–1.97)		9 (3.0%)	0.90 (0.33–2.45)		0.98 (0.34–2.78)	
Least disadvantaged (referent)	12 (5.7%)					17 (8.0%)					7 (3.3%)				
First pregnancy			0.57		0.59			0.13		0.55			0.83		0.63
Yes	27 (7.2%)	0.87 (0.52–1.43)		0.86 (0.50–1.48)		39 (10.3%)	1.44 (0.90–2.30)		1.17 (0.69–1.98)		15 (4.0%)	1.08 (0.54–2.15)		0.83 (0.38–1.78)	
No (referent)	42 (8.2%)					38 (7.4%)					19 (3.7%)				
Pregnancy risk level			0.52		0.95			0.84		0.33			0.29		0.84
Low risk pregnancy	35 (8.4%)	1.18 (0.72–1.92)		1.02 (0.54–1.94)		37 (8.9%)	1.05 (0.66–1.67)		0.74 (0.41–1.35)		19 (4.6%)	1.45 (0.73–2.89)		0.92 (0.39–2.17)	
High risk pregnancy (referent)	34 (7.2%)					40 (8.5%)					15 (3.2%)				
Consumed alcohol since pregnancy recognition			0.33		0.63			0.91		0.26			0.85		0.30
Yes	5 (5.2%)	0.63 (0.25–1.60)		0.79 (0.30–2.06)		8 (8.3%)	0.96 (0.44–2.05)		1.60 (0.71–3.60)		4 (4.2%)	1.11 (0.38–3.21)		1.83 (0.59–5.70)	
No (referent)	64 (8.1%)					69 (8.7%)					30 (3.8%)				
Antenatal service location			0.78		0.85			0.41		0.68			0.48		0.82
Regional or rural	12 (8.3%)	1.10 (0.57–2.10)		0.93 (0.44–1.97)		15 (10.4%)	1.28 (0.71–2.32)		1.17 (0.56–2.41)		7 (4.9%)	1.36 (0.58–3.19)		1.12 (0.41–3.08)	
Major city (referent)	57 (7.6%)					62 (8.3%)					27 (3.6%)				
Provider/s seen in antenatal visit			0.27		0.73			< 0.01		0.03			0.11		0.48
Midwife only	39 (8.4%)	1.26 (0.65–2.47)		1.20 (0.52–2.77)		44 (9.4%)	2.55 (1.13–5.77)		2.72 (1.07–6.90)		22 (4.7%)	2.89 (0.85–9.78)		2.70 (0.67–10.84)	
Midwife and doctor	13 (6.1%)	0.89 (0.40–2.01)		0.87 (0.38–1.99)		18 (8.4%)	2.24 (0.92–5.50)		2.37 (0.95–5.95)		6 (2.8%)	1.68 (0.41–6.83)		1.71 (0.41–7.12)	
Other provider involved	5 (15.6%)	2.56 (0.84–7.85)		1.64 (0.48–5.63)		8 (25.0%)	8.14 (2.71–24.48)		6.17 (1.83–20.82)		3 (9.4%)	6.03 (1.16–31.35)		3.35 (0.54–20.60)	
Doctor only (referent)	12 (6.7%)					7 (3.9%)					3 (1.7%)				

Adjusting for all characteristics, identifying as Aboriginal or Torres Strait Islander origin significantly increased the odds of receiving assessment (OR = 2.70; 95% CI: 1.23, 5.92), complete care (OR = 2.40; 95% CI: 1.15, 5.04) and all guideline elements (OR = 3.17; 95% CI: 1.22, 8.24) at subsequent antenatal visits. Being younger also significantly increased the odds of reporting receipt of complete care (OR = 0.91; 95% CI: 0.86, 0.96) and all guideline elements (OR = 0.91; 95% CI: 0.84, 0.99) at subsequent antenatal visits.

### Acceptability of receiving assessment and care for maternal alcohol consumption in antenatal visits

Participants reported high levels of acceptability for their alcohol consumption to be assessed (98.7%) in antenatal visits, with 88.3% of participants agreeing that it was acceptable to have their alcohol consumption assessed on multiple occasions throughout pregnancy. Reported acceptability of being provided with advice that it is safest not to consume alcohol during pregnancy (98.6%) and of the potential risks (99.3%) was also high. Almost all women reported that it would be acceptable for a referral to be offered to telephone counselling (99.0%) or a drug and alcohol service (99.4%) for further support for alcohol consumption if required. Care was also found to be highly acceptable among women who reported that they had consumed alcohol since pregnancy recognition. Of these women: 96.2% reported that they would find assessment acceptable; 82.7% that assessment on multiple occasions was acceptable; 92.5% that being provided with advice that it is safest not to consume alcohol during pregnancy was acceptable; 99.3% that being provided with advice on potential risks was acceptable; 98.5% that being offered a referral to a telephone counselling service was acceptable; and 99.3% that being offered a referral to a drug and alcohol service was acceptable.

## Discussion

This is the first comprehensive study of pregnant women’s reported receipt and acceptability of guideline recommended care for alcohol consumption and the factors associated with receiving such care in the Australian public antenatal care setting. At the initial antenatal visit, less than two thirds of pregnant women reported having their alcohol consumption assessed according to guidelines and approximately one third reported receiving the appropriate care (advice and referral) for their self-reported alcohol risk level. Most women reported that they did not receive assessment and care at subsequent antenatal visits. Reported receipt of all guideline elements were significantly associated with specific characteristics of pregnant women and/or antenatal services, including age, Aboriginal origin, education attainment level, first pregnancy, pregnancy risk level and the antenatal service location and provider seen, indicating that recommended care was not undertaken routinely for all women. A high proportion of pregnant women agreed that the provision of such assessment and care in their antenatal visits is acceptable, including those women who reported consuming alcohol since pregnancy recognition. These findings suggest that antenatal clinical guideline recommendations are currently not being universally provided and that additional strategies are required to ensure all women routinely receive the appropriate care for alcohol consumption during pregnancy [62].

A higher proportion of pregnant women reported being asked about their alcohol consumption than reported being specifically asked questions consistent with the AUDIT-C [56] (recommended validated tool). These findings are consistent with a Norwegian study that found most midwives asked about alcohol consumption, but less than half did so using a validated assessment tool [40]. The AUDIT-C assessment tool was included in the electronical medical record system used by antenatal care providers at the time of the study. Therefore, while it is possible that a small proportion of antenatal care providers may have assessed alcohol consumption using an assessment tool other than the AUDIT-C, it is potentially more likely that the assessment questions are not being asked as intended [40]. These results suggest that although system prompts may be beneficial in supporting antenatal clinicians use validated assessment tools, as a single support strategy they are unlikely to be sufficient in improving assessment according to guidelines. Further implementation support may be required to ensure that alcohol consumption behaviours are accurately identified so that care can be provided that is appropriate to a woman’s level of alcohol consumption risk [19–22].

Broadly, the study found lower rates of antenatal care provision regarding maternal alcohol consumption than previous studies in this setting. Lower reported rates of advice that it is safest not to consume alcohol during pregnancy (63.2% initial visit; 15.8% subsequent visits) were found in this study compared to all previous studies that were conducted after the release of abstinence based guidelines for pregnant women in Australia (97–99%) [39, 42]. The study also found a lower prevalence of reported referral of women who reported consuming alcohol at medium or high risk levels since pregnancy recognition (50.0% initial visit; 0.0% subsequent visits) compared to a previous study of Norwegian midwives (66% initial visit) [40]. A possible explanation for these different findings may be different populations, the use of different health systems and/or different data collection methods. The previous studies used clinician self-report, which is more susceptible to response bias and an overestimate of the prevalence of care provision [44] than data collected from client surveys, which may produce more conservative results. These results suggest that antenatal services may benefit from comprehensive implementation support, such as educational meetings and materials [63, 64], electronic prompts and reminders [65], local opinion leaders [66–68], audit and feedback [69], academic detailing [70, 71], performance monitoring [72] and leadership [66] to provide guideline recommended care for alcohol consumption during pregnancy.

Reported receipt of care was found to differ between the initial and subsequent antenatal visits and each of the elements were found to vary within the visit type. The findings related to high prevalence of care at the initial antenatal visit are similar to a Norwegian study, which reported that 97% of midwives mostly or always ask about alcohol at the initial antenatal visit and 66% mostly or always provide a referral when risky alcohol consumption is identified [40]. Further, the finding that most women at the initial antenatal visit are assessed for alcohol consumption, whereas only a small proportion receive any advice or referral related to alcohol consumption, is supported by numerous studies examining the prevalence of antenatal care for alcohol consumption during pregnancy [38, 40, 43] and literature from health care settings more broadly [45]. There are a number of potential reasons for lower prevalence of care in response to assessment at the initial antenatal visit, including that the focus of such visits is on comprehensive assessment and history taking and that there is often a lack of formalised care pathways [73]. In addition, repeat assessment and care at subsequent antenatal visits for risk factors that were not identified in the initial antenatal visit are often not prioritised even though patterns of alcohol consumption can change throughout pregnancy and rapport may need to be built over numerous visits in order for women to feel comfortable to disclose alcohol consumption [10]. Support provided to clinicians to improve adherence to guideline recommended care should focus on formalising care pathways in response to assessment and increasing care at subsequent antenatal visits.

A number of maternal and service characteristics were found to be associated with the receipt of recommended care for alcohol consumption during pregnancy, indicating that such care was not provided routinely to all women, by all antenatal care providers. At the initial antenatal visit, women attending antenatal care at a regional/rural location were more likely to report receiving all guideline elements, which is consistent with the findings by Davis et al. [27] and studies examining receipt of care for behavioural risk factors in other health care settings [53]. Pregnant women’s reported alcohol consumption since pregnancy recognition was not found to be associated with the provision of assessment and care at subsequent antenatal visits, which is also consistent with previous research [38, 47]. The characteristics that were found to be associated with reported receipt of all guideline elements at subsequent antenatal visits were being younger or being of Aboriginal and Torres Strait Islander origin. These characteristics are similar to those previously reported in a large study conducted with postpartum women in the United States [34] and consistent with qualitative interviews with Australian midwives regarding decision processes for addressing alcohol consumption at subsequent antenatal visits [33]. Although it is unknown whether the Aboriginal women who participated in the study were different in any way to those who did not, such findings suggest that decision making processes regarding assessment and care for alcohol consumption undertaken at subsequent visits could be based on stereotypes regarding alcohol consumption [34] rather than being directed by universal guideline recommendations. System changes, such as education of antenatal care providers to address stereotypes [74] and electronic prompts reiterating universal screening recommendations [65, 75], may support antenatal care providers deliver care routinely to all pregnant women.

There was a high level of acceptability among pregnant women for all elements of guideline recommended care for addressing alcohol consumption during pregnancy. These findings extend that of a previous Australian study that found a high level of acceptability for being asked about alcohol consumption and being advised not to consume alcohol during pregnancy among women generally [51]. These findings are also consistent with previous literature [12, 36, 38, 76, 77], which suggests that pregnant women perceive their antenatal care providers as an important source of information for making informed choices about alcohol consumption during pregnancy. Such findings suggest that barriers to care provision previously reported by antenatal care providers, such as addressing alcohol will cause discomfort [39, 40, 78, 79] and impact on the client-clinician relationship [80–82], may be unfounded. Further research is required to confirm pregnant women’s acceptability of guideline elements for maternal alcohol consumption post receipt of such care as direct experience may change perceptions of acceptability [83].

The results of this study should be considered in light of a number of its methodological strengths and limitations. The study was conducted with a large sample of randomly selected pregnant women who completed the survey within 4 weeks of their antenatal visit to limit any potential recall bias. The study is one of few to have utilised pregnant women’s self-report, which is reported to limit response bias that may affect health providers’ report of care provision [44]. Pregnant women’s self-reported alcohol consumption since pregnancy recognition was used to assess whether the antenatal care provider asked the AUDIT-C questions and offered referrals appropriately, however, women may not have provided the same information about their alcohol consumption to their antenatal care provider. As only a small number of respondents reported consuming alcohol at medium and high risk levels, potential associations with receipt of referral could not be examined. This may have been due to women underreporting the level of alcohol they are consuming during pregnancy, which has been reported by previous studies [30, 34]. Further research is required to identify factors associated with women accurately reporting their level of alcohol consumption during their pregnancy, as accurate reporting of risk is required in order for appropriate support to be offered to assist women abstaining from alcohol during pregnancy. For ethical reasons, the study did not collect information on previous live births and stillbirths and, as such, a woman’s first pregnancy is not specifically defined as either parity or gravidity. Lastly, the study was limited to pregnant women aged 18 years and over who were proficient in English, had not experienced an adverse pregnancy outcome (miscarriage or stillbirth) and were receiving the majority of their antenatal care through a public antenatal service within one health district in Australia. Therefore, the extent to which these findings generalise to other women and antenatal services in Australia and internationally is unknown.

## Conclusions

The results of this study indicate that although assessment and care for maternal alcohol consumption is highly acceptable to pregnant women, receipt of such care in public antenatal services is suboptimal and preferentially provided based on the characteristics of pregnant women and antenatal services. Opportunities exist to increase provision of clinical guideline recommended care in public antenatal services through the implementation of comprehensive support strategies for antenatal care providers. Future research is required to investigate the effectiveness of such implementation strategies and their acceptability to antenatal care providers.

## Data Availability

The datasets used and analysed during the current study are available from the corresponding authors on reasonable request.

## Abbreviations

AMIHS: Aboriginal Maternal Infant Health Service
AUDIT-C: Alcohol Use Disorders Identification Test – Consumption
CATI: Computer assisted telephone interview
